# Bioaminergic signaling in parasitic flatworms: a perspective for linking GPCRs to behavior and drug discovery

**DOI:** 10.3389/fpara.2026.1933406

**Published:** 2026-09-23

**Authors:** Erika Bistran, M. Evelyn Carpenter, Daniel J. Sprague

**Affiliations:** 1 Department of Biochemistry and Molecular Biology, Medical University of South Carolina, Charleston, SC, United States; 2 Hollings Cancer Center, Medical University of South Carolina, Charleston, SC, United States

**Keywords:** anthelmintics, cestode, dopamine, drug discovery, fluke, histamine, schistosome, serotonin

## Abstract

Bioaminergic signaling regulates behaviors that are central to parasite survival, including movement, attachment, feeding, pairing, and reproduction, yet the molecular pathways linking bioamines to these phenotypes remain incompletely defined in parasitic flatworms. In this Perspective, we propose a pathway-to-phenotype framework for organizing future studies of flatworm aminergic signaling. This framework links six interconnected areas of investigation: the bioamine ligand, the receptor or transporter that detects or clears it, the downstream signaling pathway, the relevant cell type or tissue, the experimental perturbation used to test function, and the resulting parasite phenotype. Serotonin signaling through Sm5HTR in *Schistosoma mansoni* provides the clearest current example, connecting receptor activation to cAMP signaling, nervous-system localization, and movement. By contrast, dopaminergic, histaminergic, octopaminergic, tyraminergic, transporter-associated, and non-schistosome flatworm systems remain less completely mapped. We highlight these and other gaps, and discuss how comparative functional profiling across parasite lineages could reveal conserved signaling principles, parasite-selective pharmacology, and new opportunities for anthelmintic discovery.

## 1 Introduction

For parasitic flatworms, adaptable behavior is essential for survival: movement, attachment, feeding, pairing, and reproduction all determine whether a worm can persist in a host. Adult schistosomes must maintain their life within the vasculature, pair, feed, and reproduce (Ross et al., 2002; Colley et al., 2014). Liver flukes must do the same, migrating through host tissues and establishing long-term residence in the liver, bile ducts, or associated hepatobiliary niches (Andrews, 1999). Cestodes must attach to host tissues or persist in cystic forms while coordinating growth and reproductive maturation (Sapp and Bradbury, 2020; Jeon and Eom, 2024). These processes depend on neuromuscular and neuroendocrine signaling systems that remain incompletely understood, but are increasingly able to be studied through genomic, pharmacological, and phenotypic tools (Pax et al., 1996a; Preza et al., 2018).

Bioaminergic signaling offers a practical way into studying this biology because it connects recognizable neurotransmitters to receptors, second messengers, and measurable parasite behaviors (McVeigh et al., 2018b). Serotonin, dopamine, histamine, tyramine, and octopamine have all been implicated in flatworm neuromuscular control and parasite physiology (Tomosky et al., 1974; Mellin et al., 1983; Ribeiro and Webb, 1983; Pax et al., 1984; Eriksson et al., 1996; Pax et al., 1996b; Boyle et al., 2000; Hrckova et al., 2002; Ribeiro et al., 2005; Brooker et al., 2011; Ribeiro et al., 2012; Ribeiro, 2015; El-Sakkary et al., 2018; Herz and Brehm, 2021; Robb et al., 2026). Historically, the field asked whether these neurotransmitters were present and whether exogenous compounds altered movement. Those questions were necessary, but they now would benefit from complementation with in-depth mechanistic approaches. The central question now is how specific aminergic receptors and transporters connect conserved neurotransmitters to parasite signaling pathways and phenotypes.

This Perspective proposes that the study of bioaminergic signaling in parasitic flatworms can be organized around a pathway-to-phenotype framework. In this model, mechanistic understanding emerges by connecting six areas of investigation: the bioamine ligand, the receptor or transporter that detects or clears it, the second messenger pathway engaged, the cell type or tissue in which signaling occurs, the experimental perturbation used to test receptor or ligand function, and the parasite phenotype that results. This reframing moves the field beyond catalogs of neurotransmitters and predicted receptors toward mechanistic maps that can support both parasite neurobiology and anthelmintic discovery (**Figure 1**). We think that this framework is especially timely because increasingly well-assembled flatworm genomes (Stevens and Doyle, 2026) have revealed many candidate signaling proteins, and functional studies have begun to show that parasite aminergic receptors may diverge pharmacologically and mechanistically from host systems (McVeigh et al., 2018a).

**Figure 1:**
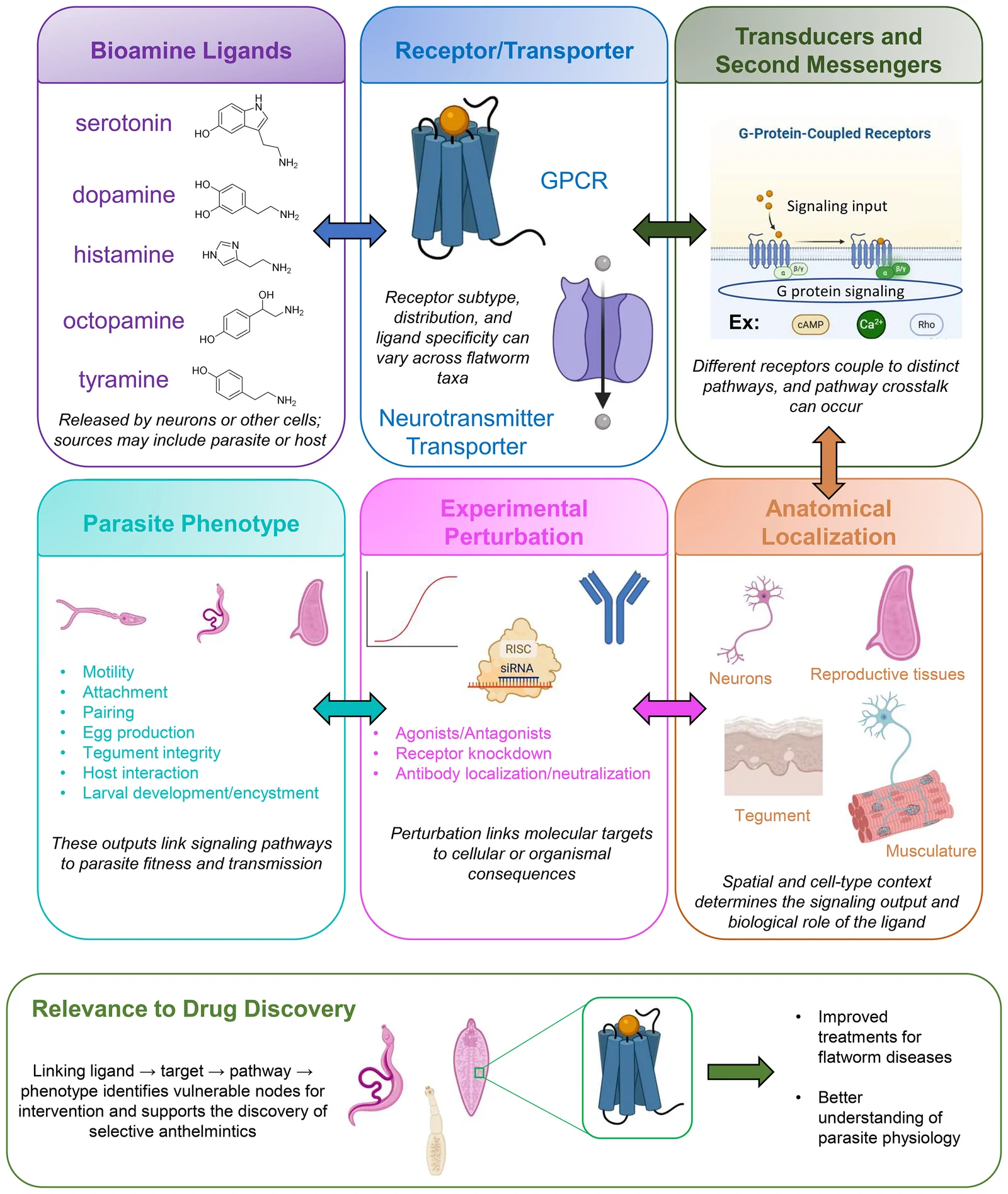
A pathway-to-phenotype framework for functional profiling of bioaminergic signaling in parasitic flatworms. The framework comprises six interconnected areas of investigation: the bioamine ligand (purple), its receptor (blue), the transducer network and second-messenger signaling (olive), the anatomical localization of signaling components (orange), experimental approaches used to interrogate pathway function (pink), and the resulting parasite phenotypes (teal). These areas of investigation are neither sequential steps nor prerequisites for entry; investigations may begin in any domain for which reproducible evidence is available. Bidirectional arrows represent the iterative movement between domains as hypotheses are generated, tested, and refined. By integrating evidence across these areas, the framework supports increasingly resolved and mechanistically grounded models linking bioaminergic signaling to parasite physiology and behavior.

Here, we briefly review the prior literature, presenting schistosome serotonin signaling as the clearest current example of this approach. We then consider less complete but informative dopaminergic, histaminergic, octopaminergic, and tyraminergic systems. Finally, we broaden the discussion to cestodes and other trematodes, highlight transporters as an understudied layer of signaling, and propose criteria for future functional profiling studies.

## 2 Schistosomes

### 2.1 Sm5HTR as the benchmark for functional profiling

The strongest current example of pathway-to-phenotype mapping of bioaminergic pathways in parasitic flatworms is serotonergic signaling through Sm5HTR in *Schistosoma mansoni* (Pax et al., 1996a; Chan et al., 2016a; Chan et al., 2016b; Chan et al., 2018; Marchant et al., 2018). Serotonin has long been recognized as myoexcitatory in schistosomes, stimulating motor activity and potentiating body wall muscle contraction (Pax et al., 1996a). The characterization of Sm5HTR transformed that observation into a mechanistic pathway.

Sm5HTR was identified as a serotonin-responsive GPCR distantly related to human 5-HT_7_ receptors. In heterologous expression systems, the receptor responded to serotonin and increased intracellular cAMP, consistent with a 5-HT_7_-like Gα_s_-coupled signaling mechanism. Immunolocalization revealed Sm5HTR to be broadly located within the parasite nervous system, including the cerebral ganglia, longitudinal nerve cords, peripheral innervation of body wall muscle, and tegument-associated neural elements. RNA interference (RNAi) in larvae and adults caused marked hypoactivity and reduced responsiveness to exogenous serotonin (Pax et al., 1996a). Screening efforts also discovered new agonists and antagonists for this receptor and efforts were also made to delineate schistosome specific from human-specific ligands, ultimately setting up Sm5HTR for future studies in drug discovery (Chan et al., 2016a; Chan et al., 2016b; Chan et al., 2018; Marchant et al., 2018).

Altogether, studies of Sm5HTR connect serotonin as the ligand, Sm5HTR as the receptor, cAMP as the signaling output, the nervous system as the anatomical context, and movement as the measurable phenotype. Sm5HTR therefore provides a useful benchmark for the comprehensive study of bioaminergic signaling in parasitic flatworms.

### 2.2 Dopamine, histamine, and octopamine: useful, but incomplete maps

#### 2.2.1 Dopamine

Other aminergic receptors provide important, but less complete, examples of bioaminergic signaling in schistosomes. Dopaminergic signaling in schistosomes is supported by both SmD2 (Taman and Ribeiro, 2009) and SmGPR-3 (El-Shehabi et al., 2012). Both receptors are putative dopamine-activated GPCRs from *S. mansoni* that are associated with the parasite neuromusculature. The ligands of these receptors were identified using yeast-based functional reporter systems, and SmD2 was also studied using heterologous expression in mammalian cells. However, dopamine signaling has not been mapped as completely as serotonin signaling. What remains missing among dopamine-responsive receptors, which has been established Sm5HTR is comprised of the following: receptor activation, pathway coupling, localization, perturbation, and phenotype.

Dopamine signaling in schistosomes also brings up another key point: flatworm receptors should not be forced into vertebrate categories without functional evidence. Parasite receptors may display divergent ligand sensitivity, unusual pharmacology, or atypical coupling. These two putative dopamine receptors are great examples of this. SmGPR-3 shows a mixed pharmacological profile: an *antagonist* of a mammalian dopamine receptor was an *agonist* of this receptor (El-Shehabi et al., 2012). Likewise, by sequence, SmD2 is closely related to human D_2_ receptors (D_2_R). However, the D_2_R family of receptors are Gα_i/o_-coupled (Jiang et al., 2001) while SmD2 seems to be Gα_s_ coupled. This is one reason dopaminergic signaling is particularly interesting: it may preserve enough similarity to established catecholaminergic biology to guide discovery, while diverging enough to create parasite-selective pharmacological opportunities.

#### 2.2.2 Histamine

Histaminergic signaling is important because it demonstrates that flatworm aminergic GPCRs do not all converge on Gα_s_/Gα_i_-mediated cAMP signaling. A histamine-responsive *S. mansoni* GPCR (SmGPCR, later renamed SmGPR-1), which is mostly expressed in the body wall muscles, has been associated with intracellular calcium mobilization rather than cAMP accumulation (Hamdan et al., 2002). This suggests that a Gα_q_-like pathway is at play, and has a practical implication: receptor screens based solely on cAMP will miss or misclassify calcium-linked aminergic receptors. Histamine is also conceptually interesting because parasites may encounter both endogenous and host-derived histamine, particularly in inflammatory environments (Kourilova et al., 2004; Ribeiro, 2015). Whether histamine signaling primarily regulates movement, tegument physiology, host sensing, or immune-interface biology remains unresolved. This makes histamine a useful reminder that aminergic signaling in parasitic flatworms may not be limited to classical neurotransmission. Some pathways may also participate in sensing or responding to host-derived chemical cues.

SmGPR-2 was the second histamine-responsive GPCR profiled from *S. mansoni* (El-Shehabi and Ribeiro, 2010). SmGPR-2 is uniquely upregulated in schistosomula, and is expressed in the peripheral nervous system (specifically, in the subtegumental nerve plexus). SmGPR-2 has a unique orthosteric binding pocket and pharmacology when compared with mammalian histamine receptors. For example, the canonically conserved aspartate, D^3.32^ [Ballesteros-Weinstein residue numbering convention (Ballesteros and Weinstein, 1995; Isberg et al., 2015)], is an asparagine in the transmembrane helix 3 (TM3) of SmGPR-2. The study demonstrated that many mammalian histamine receptor antagonists were ineffective on the schistosome receptor. It is well accepted that D^3.32^ is essential for the activation of most Class A GPCRs by their cognate ligands (Leurs et al., 2009; McCorvy and Roth, 2015), and this is likely the reason for the interesting divergent pharmacology seen in the original report by El-Shehabi and Ribeiro. Overall, early studies on histamine signaling in flatworms are interesting and informative, and much more work still remains to be done.

#### 2.2.3 Octopamine and tyramine

Octopamine and tyramine may offer the most parasite-selective opportunities for drug discovery because they are prominent in many invertebrate neuromodulatory systems but are less central to mammalian host neurobiology (Dougan and Wade, 1978; Burrows and Pfluger, 1995; Erspamer, 2009). The importance of octopamine signaling in *S. mansoni* has gained anatomical, phenotypic, and pharmacological support (El-Sakkary et al., 2018). Octopamine labeling is widespread in adult worms and schistosomula, and octopamine-associated studies have refined our understanding of the schistosome nervous system.

Octopamine, and related tyrosine-derived compounds modulate schistosomulum motility and length, and focused drug screening efforts have identified analogs of these chemotypes that alter parasite movement and morphology (El-Sakkary et al., 2018). However, a receptor-level map remains incomplete. The field still needs to identify the specific tyramine and octopamine receptors responsible for these effects and determine whether they signal through cAMP, calcium, inhibitory pathways (e.g., the Gα_i_/_o_ family of Gα proteins), or biased mechanisms. To date, no such receptor has been functionally profiled.

Together, these studies show that bioaminergic signaling in flatworms is not a single pathway, but a set of related signaling architectures at different stages of functional resolution. Serotonin provides the clearest pathway-to-phenotype map, linking receptor activation to cAMP signaling, nervous-system localization, and movement. Histamine illustrates calcium-linked receptor signaling; dopamine points to aminergic modulation of neural circuits; and octopamine and tyramine suggest invertebrate-like neuromodulatory systems with possible parasite-selective potential. However, that selectivity remains hypothetical until the relevant parasite receptors are deorphanized and compared pharmacologically with host aminergic receptors and other potential off-targets.

## 3 Cestodes and other flukes

Up to this point, the functional characterization of bioaminergic GPCRs in parasitic flatworms has focused heavily on schistosome neurobiology, but bioaminergic signaling should not be treated as a schistosome-specific topic. Cestodes (e.g., *Echinococcus* spp. and *Mesocestoides* spp.) and other trematodes such as *Fasciola hepatica* and *Clonorchis sinensis* provide essential comparative context because they occupy different host niches, rely on different attachment strategies, and undergo distinct developmental and reproductive programs. We argue that a schistosome-centric literature may make it difficult to distinguish general flatworm principles from lineage-specific biology.

Cestode serotonergic GPCRs provide an important example of bioaminergic signaling in parasitic flatworms (Camicia et al., 2018; Camicia et al., 2021; Kreshchenko et al., 2021). Serotonin-responsive GPCRs from *Echinococcus* and *Mesocestoides* species have been cloned and functionally characterized (Camicia et al., 2018; Camicia et al., 2021). One set of these receptors cluster phylogenetically with 5-HT_1_-like receptors and seem to represent a unique new class of parasite GPCRs (Camicia et al., 2021). Another set cluster phylogenetically with 5-HT_7_-like GPCRs (Camicia et al., 2018). The 5-HT_7_-like receptors stimulate cAMP production in response to serotonin, and show distinctive pharmacological properties. The 5-HT_1_-like receptors were deorphanized using a promiscuous Gα_15_ effector protein and Ca^2+^-flux assays, and so future work will need to be performed to determine the endogenous coupling partner.

Serotonin also increases larval motility in cestode models, and localization of these aforementioned serotonergic receptors in *E. granulosus* larvae has been reported near the suckers (Camicia et al., 2021). These observations suggest that serotonergic signaling may influence not only movement but also attachment-related biology in tapeworms.

This comparison is important for two reasons. First, it demonstrates that conserved neurotransmitters can operate through parasite-adapted receptors across flatworm lineages. Second, it provides a practical route to identifying which features of aminergic signaling are broadly conserved and which are lineage-specific. A receptor that is functionally conserved across schistosomes and cestodes may represent a broad-spectrum target class. By contrast, a receptor or pathway restricted to a particular parasite group may provide opportunities for selective intervention. We expect these differences to come to light as the field continues to rapidly make new discoveries.

Studies in other trematodes, including liver flukes, remain comparatively underdeveloped. The Maule group has made pivotal advances in our understanding of the role of serotonergic systems in *F. hepatica* (Robb et al., 2026), including localization of several receptors within the worm and RNAi studies to determine the *in vivo* function of serotonin receptors and transporters from *F. hepatica*. However, complete pharmacological profiling of the corresponding serotonin receptors has not yet been accomplished. Likewise, dopaminergic antagonists have been shown to modulate the reproductive behavior and chemotaxis of the carcinogenic liver fluke *C. sinensis* (Li et al., 2019; Dai et al., 2020); to date no bioaminergic receptor has been cloned and profiled from this fluke. However, both of these organisms are biologically important, and occupy host environments distinct from the vasculature in which one finds blood flukes. Expanding functional profiling into these systems could reveal whether aminergic signaling regulates migration, tissue residence, feeding, attachment, or reproductive output in ways that differ from schistosomes. This comparative work is extremely exciting, and will be essential to study within the field-wide framework of parasite neurobiology.

## 4 Transporters: the underdeveloped half of aminergic signaling

Aminergic signaling is often framed around receptors, but neurotransmitter transporters are equally important (Sitte et al., 2025). Among their various roles, neurotransmitter transporters regulate extracellular neurotransmitter availability, signal duration, and receptor desensitization. In schistosomes, serotonin and other amine transporter-like proteins have been identified and discussed as possible drug targets (Patocka and Ribeiro, 2007; Ribeiro and Patocka, 2013). A recent study by the Maule group also identified a serotonin transporter in *F. hepatica* (Robb et al., 2026). A dopamine transporter has also been identified in *C. sinensis* (Lee et al., 2025). However, transporter biology in parasitic flatworms remains much less developed than corresponding receptor biology.

This remains an important opportunity. Transporter inhibition can reshape signaling across *multiple* receptors by changing ligand availability rather than directly targeting a *single* receptor. In a parasite neuromuscular network, this could disrupt movement, attachment, or behavioral state through multiple receptors simultaneously. Functional profiling of transporters should include substrate uptake assays (Aggarwal and Mortensen, 2017), inhibitor sensitivity (Glatfelter et al., 2026), spatial and life-stage expression (Wendt et al., 2021), and whole-parasite phenotyping (Patocka and Ribeiro, 2007; Ribeiro and Patocka, 2013). Transporter inhibitors should also be paired with receptor antagonists to determine which receptors mediate downstream phenotypes. As with receptors, however, transporter-directed strategies will require careful selectivity profiling because host monoamine transporters are pharmacologically sensitive and physiologically important [e.g., the target of the antidepressant selective serotonin reuptake inhibitors (SSRIs)] (Boschloo et al., 2023). Transporters may also be useful for distinguishing endogenous parasite signaling from responses to host-derived ligands. If a parasite expresses transporters capable of clearing serotonin, dopamine, or related amines from its local environment, this could shape how the worm responds to both internally produced and host-derived signals. In this sense, transporters are not merely terminators of synaptic signaling; they may help define the chemical interface between parasite and host.

Taken together, these examples above show how far the field has come, but also where the gaps remain. Serotonin signaling, especially in schistosomes and cestodes, has begun to connect receptors, second messengers, anatomy, and parasite behavior. For dopamine, histamine, octopamine, tyramine, and bioamine transporters, the evidence is promising but less complete. In many cases, the links between ligand, molecular target, signaling pathway, and parasite phenotype are still missing. This is the rationale for our proposed pathway-to-phenotype framework (**Figure 1**, **Table 1**).

**Table 1:** Maturity of the field with regard to functional profiling of bioaminergic signaling in parasitic flatworms.

System	Pathway	Best-supported example	Current strength of evidence	Key gap
Schistosomes	Serotonin	Sm5HTR in *S. mansoni*	Strong pathway-to-phenotype evidence: serotonin responsiveness, receptor identity, cAMP signaling, nervous-system localization, RNAi phenotype, and movement response are linked in one system.	Broader testing across life stages and non-motility phenotypes, including pairing, attachment, egg production, tegument function, and host interaction.
	Dopamine	SmD2 and SmGPR-3 in *S. mansoni*	Dopamine-responsive GPCRs identified; receptors are widely expressed in the nervous system, supporting catecholaminergic signaling in schistosomes.	Key unresolved connections include native coupling, perturbation studies, and parasite-level phenotypes.
	Histamine	SmGPR-1 and SmGPR-2	Key evidence that flatworm aminergic GPCRs can engage calcium-linked signaling rather than just cAMP pathways, and that orthosteric binding pockets may vary in lower metazoans when compared to mammalian counterparts.	The physiological role of histaminergic signaling remains unclear, including whether signaling reflects endogenous neurotransmission, host-signal sensing, or both.
	Octopamine/Tyramine	There is octopamine-associated signaling in *S. mansoni*	Anatomical, phenotypic, and pharmacological evidence supports a role in schistosome neuromuscular regulation.	Specific tyramine/octopamine receptors remain to be identified, deorphanized, and linked to signaling pathways and phenotypes
	Bioamine Transporters	Serotonin and other amine transporter-like proteins	Candidate regulators of extracellular bioamine availability and potential drug targets	Substrate specificity, inhibitor sensitivity, tissue localization, life-stage expression, and whole-parasite consequences upon perturbation remain underdeveloped
Cestodes	Serotonin	5-HT GPCRs from *E. granulosus* and *M. corti*	Functionally characterized receptors show serotonin responsiveness, cAMP signaling, distinctive pharmacology, and links to larval motility.	Expanded receptor-to-phenotype validation across cestode species, stages, tissues, and attachment-related behaviors.
Non-schistosome Trematodes	Serotonin and Dopamine	Serotonergic signaling in *F. hepatica*; dopaminergic signaling in *C. sinensis*	Evidence supports roles for aminergic signaling in non-schistosome trematodes, including neuromuscular, behavioral, and reproductive regulation.	Corresponding receptors remain to be characterized functionally; receptor cloning, deorphanization, pathway coupling, and integrated receptor-to-phenotype mapping are needed.

## 5 A functional profiling framework for the next phase of discovery

Here we present a standardized framework in which to study parasitic flatworm bioaminergic signaling. A predicted GPCR should not be treated as a serotonin, dopamine, histamine, tyramine, or octopamine receptor solely because of sequence similarity. In reality, while transmembrane homology within GPCRs is fairly high (>70% in some cases), total identity and homology is low for *Platyhelminth* GPCRs when compared with higher metazoa. Likewise, a motility response to a bioamine should not be interpreted as evidence for a specific GPCR without pathway-level support. There could be numerous signaling pathways at work.

Thus, we propose that future studies organize aminergic signaling around several questions:

The ligand: Which bioamine activates or regulates the pathway?

The molecular target: Which receptor or transporter detects, clears, or modulates the ligand?

The pathway: Does signaling involve cAMP, calcium, β-arrestin, ion-channel modulation, or another route?

The cell type: Where is the receptor or transporter expressed in the parasite?

The method of perturbation for the study: What happens after RNAi, pharmacological inhibition, overactivation, or transporter blockade?

The ultimate phenotype: Does the pathway affect movement, attachment, pairing, feeding, egg output, survival, or host interaction?

Importantly, these six areas of investigation represent interconnected forms of evidence rather than a required experimental sequence. A study does not need to begin with a cloned or deorphanized receptor, and the absence of receptor-level information should not prevent investigation of a signaling system. Any reproducible observation—including detection of a bioamine, a ligand- or compound-induced phenotype, the expression or localization of a candidate receptor, or a measurable signaling response—can provide an initial evidence anchor. Investigators can then use that anchor to identify plausible connections and select the experiment that most clearly distinguishes among them. Each experimentally supported connection increases the resolution of the pathway model; no individual study is expected to address every domain.

Systems with limited baseline information can therefore be approached through several complementary entry points. A phenotype-first strategy may begin with quantitative profiling of parasite responses to a bioamine or focused compound panel, followed by the use of transcriptomic and spatial-expression data to prioritize candidate receptors in the responsive life stage, tissue, or cell population. A candidate-first strategy may begin with orphan GPCRs prioritized through phylogenetic placement, receptor sequence features, lineage distribution, and expression, followed by functional screening against multiple candidate ligands and signaling readouts. A spatial-first strategy may begin with a defined neuronal, muscular, or other cell population and ask which biosynthetic enzymes, transporters, receptors, and signaling components are expressed within it. For octopamine signaling in *S. mansoni*, the existing anatomical, pharmacological, and phenotypic observations already provide an entry point from which candidate receptors can be prioritized and tested. Comparative analyses with free-living flatworms such as *Schmidtea mediterranea* may further help distinguish conserved flatworm receptor families from parasite-specific candidates (Zamanian et al., 2011). However, phylogenetic position and expression can only prioritize experimentally testable hypotheses; they cannot independently establish ligand identity, pathway coupling, or physiological function. The immediate objective is therefore not to complete the framework in a predetermined order, but to test the missing connection that will most effectively advance the system from association toward mechanism. Regardless of the initial entry point, confidence in a proposed pathway increases when independent molecular, spatial, pharmacological, and phenotypic evidence converges on the same model.

Assay design is especially important. Sm5HTR would be captured by cAMP assays. Histamine-responsive signaling may require calcium assays. Other receptors may show biased signaling or context-dependent coupling. Standard mammalian bioaminergic signaling assays, or coupling partners, may also not be sufficient to study the relevant parasite physiology and pharmacology. Therefore, future profiling should avoid single-readout classification whenever possible. Similarly, whole-parasite phenotyping should move beyond motility alone. Movement is important, but aminergic pathways may also regulate attachment, pairing, egg production, tegument function, sensory behavior, and developmental transitions.

## 6 Implications for anthelmintic discovery

Bioaminergic signaling offers several attractive features for drug discovery. GPCRs and transporters are membrane-associated, ligand-sensitive, and pharmacologically tractable (Zhang et al., 2024). Aminergic pathways regulate behaviors that are likely relevant to parasite survival. Some parasite receptors show pharmacological divergence from host receptors. Finally, receptor-based assays can be scaled for screening.

However, the choice of targets must be careful. Serotonin, dopamine, and histamine pathways are essential in mammalian hosts as well, so parasite selectivity cannot be assumed. Octopamine and tyramine systems may offer a larger selectivity window, but their receptor biology is not yet sufficiently resolved. A compound that alters parasite movement *in vitro* is promising, but the phenotype must be connected to survival, attachment, migration, reproduction, clearance, or some other essential niche to be useful as a therapeutic target. Target-based screens and whole-parasite assays should therefore be integrated, rather than treated as competing approaches.

The most promising druggable targets in the future will likely share three features: functional importance, parasite-selective pharmacology, and measurable whole-parasite consequences. Sm5HTR-like studies provide the template. The next challenge is to apply that template across the entire range of aminergic systems and parasite lineages.

## 7 What is still missing?

Several unresolved questions should define the next phase of aminergic signaling research in parasitic flatworms.

First, what intracellular signaling machinery do parasite receptors actually use? Most functional assignments rely on heterologous expression systems, where parasite receptors are tested using mammalian (or other) downstream signaling machinery. These assays are essential, but they may not fully reproduce parasite-specific G protein coupling, effector selectivity, receptor trafficking, or pathway bias. To date, functional profiling of native parasitic flatworm heterotrimeric G protein complexes remains limited. This is a major gap. If flatworm G proteins, adenylyl cyclases, phospholipases, or downstream effectors differ from mammalian counterparts, then receptor behavior in heterologous systems may only partially reflect signaling in the parasite.

Second, what are the worms sensing? Aminergic GPCRs may respond to parasite-synthesized neurotransmitters, host-derived signals, or both. This is key since flukes and tapeworms live in chemically complex environments containing host neurotransmitters, inflammatory mediators, metabolic cues, and parasite-derived signaling molecules. Histamine is a particularly interesting example because it could plausibly function as an endogenous parasite signal and as a host-associated inflammatory cue. Serotonin and dopamine may raise similar questions in particular host niches. Distinguishing autocrine or paracrine parasite signaling from host-signal detection will require careful integration of biosynthetic enzyme expression, transporter localization, ligand measurements, and physiological context.

Third, how do aminergic pathways interact with other GPCR systems? Bioaminergic signaling is likely not the entire story. Neuropeptide receptors (Marks et al., 1995; Marks et al., 1997; Li et al., 2024; Geetha et al., 2026), cholinergic receptors (MacDonald et al., 2015), and other families (McVeigh et al., 2018a) are also likely central to parasite behavior and development, and may participate in pathway ‘cross-talk’. Work on neuropeptide signaling highlights that aminergic pathways should be studied as part of broader neuromodulatory networks rather than in isolation. This network perspective may be especially important for drug discovery, where partial disruption of one pathway may be compensated by another. The six areas of investigation described here can accommodate this complexity because they are not restricted to isolated, one-ligand–one-receptor pathways. Multiple bioaminergic, neuropeptidergic, or cholinergic receptors may act through the same G protein or second messenger, be expressed within the same cell or tissue, or influence a common phenotype. Conversely, a single receptor may produce different effects depending on its anatomical location, developmental stage, or signaling context. These relationships can be investigated by first defining the signaling and phenotypic effects associated with each candidate pathway and then determining whether perturbation of one pathway alters the response produced by another. Measurements made at multiple time points can help distinguish immediate receptor-mediated signaling from later compensatory or feedback responses, while spatial and single-cell expression data can indicate whether pathway integration occurs within the same cells or through communication among different cell populations. Importantly, two pathways affecting the same second messenger or phenotype does not, by itself, demonstrate direct cross-talk, because distinct pathways may independently converge on the same biological output. Establishing such relationships therefore requires receptor-specific perturbations, combined perturbation experiments, and confirmation using complementary signaling and phenotypic measurements. The framework can thus be expanded from individual pathways into a network of experimentally supported interactions as evidence accumulates. Finally, which phenotypes matter most? Acute motility assays are useful, but they are not always sufficient. For parasites, other relevant outcomes may include attachment, tissue migration, pairing stability, egg production, tegument maintenance, host sensing, or developmental transitions (Hahnel et al., 2018; Li et al., 2024; Geetha et al., 2026). A pathway that produces only a subtle motility phenotype *in vitro* may still be essential for host persistence or transmission. Conversely, a strong movement phenotype might not translate into therapeutic efficacy unless it disrupts survival, localization, or reproduction. Resolving this distinction will be essential for translating pathway biology into therapeutic relevance.

## 8 Conclusions

Given the massive advances in genome annotations and pharmacological assay options over the past decade, the study of bioaminergic signaling in parasitic flatworms is now positioned to move beyond basic ‘cataloging’ of the presence of bioamines and predicted receptors. The field can now ask *how* these important molecules are connected to receptors, transporters, second messengers, cell types, and parasite behaviors. Sm5HTR from *S. mansoni* demonstrates the power of this approach by linking serotonin to cAMP signaling, nervous-system localization, and movement. Dopamine, histamine, octopamine, tyramine, and cestode serotonergic systems provide additional but less complete maps. Likewise, unraveling bioaminergic signaling pathways in liver flukes has a bright and important future.

A pathway-to-phenotype framework will allow for in-depth mechanistic studies and improve the possibility of using these targets for efforts in drug discovery. It will also enrich our understanding of parasite physiology. By integrating ligand identification, receptor deorphanization, transporter biology, pathway assays, spatial expression, perturbation, and parasite phenotyping, researchers can better identify which aminergic signaling systems are essential at each life cycle stage, which are druggable, and which are parasite selective. The next step for flatworm neurobiology is therefore not simply to deorphanize predicted receptors, but to determine which signaling pathways control parasite behavior, which are essential *in vivo*, and which can be selectively exploited for the development of new therapeutics. We expect the future of this field to be bright and filled with new discoveries!

## Data Availability

The original contributions presented in the study are included in the article/supplementary material. Further inquiries can be directed to the corresponding author.
